# Elevation of plasma angiotensin II level is a potential pathogenesis for the critically ill COVID-19 patients

**DOI:** 10.1186/s13054-020-03015-0

**Published:** 2020-06-05

**Authors:** Zhiyong Wu, Rui Hu, Cuizhen Zhang, Wei Ren, Anfeng Yu, Xiaoyang Zhou

**Affiliations:** 1 Department of Cardiovascular Surgery, Renmin Hospital of Wuhan University, No. 238, Jiefang Road, Wuhan, 430060 China; 2 Department of Cardiology, Renmin Hospital of Wuhan University, No. 238, Jiefang Road, Wuhan, 430060 China

**Keywords:** SARS-CoV-2, COVID-19, ACE2, Angiotensin II

Little is known about the mechanism of coronavirus disease 2019 (COVID-19)-induced critical illness and death. The host infection mediated by SARS-CoV-2 is mainly relied on ACE2 receptor. There is still a lack of clinical data about the effects of interaction of ACE2 and SARS-CoV-2 on RAS system and disease progression. We investigated the plasma angiotensin II (Ang II) and renin levels in 82 non-hypertensive patients (42 mild cases, 25 severe cases, and 15 critically ill cases) infected by SARS-CoV-2 and 12 critically ill patients not infected by SARS-CoV-2 serving as control.

Plasma Ang II level was higher than that of normal range in the majority of COVID-19 cases (90.2%), especially the plasma Ang II positive rate in the critically ill COVID-19 patients (100%). Plasma Ang II level in critically ill COVID-19 patients was significantly higher than that of control and those with mild COVID-19 symptoms (Fig. 1). Univariate analysis indicated a positive correlation between plasma Ang II level and COVID-19 severity.

**Fig. 1:**
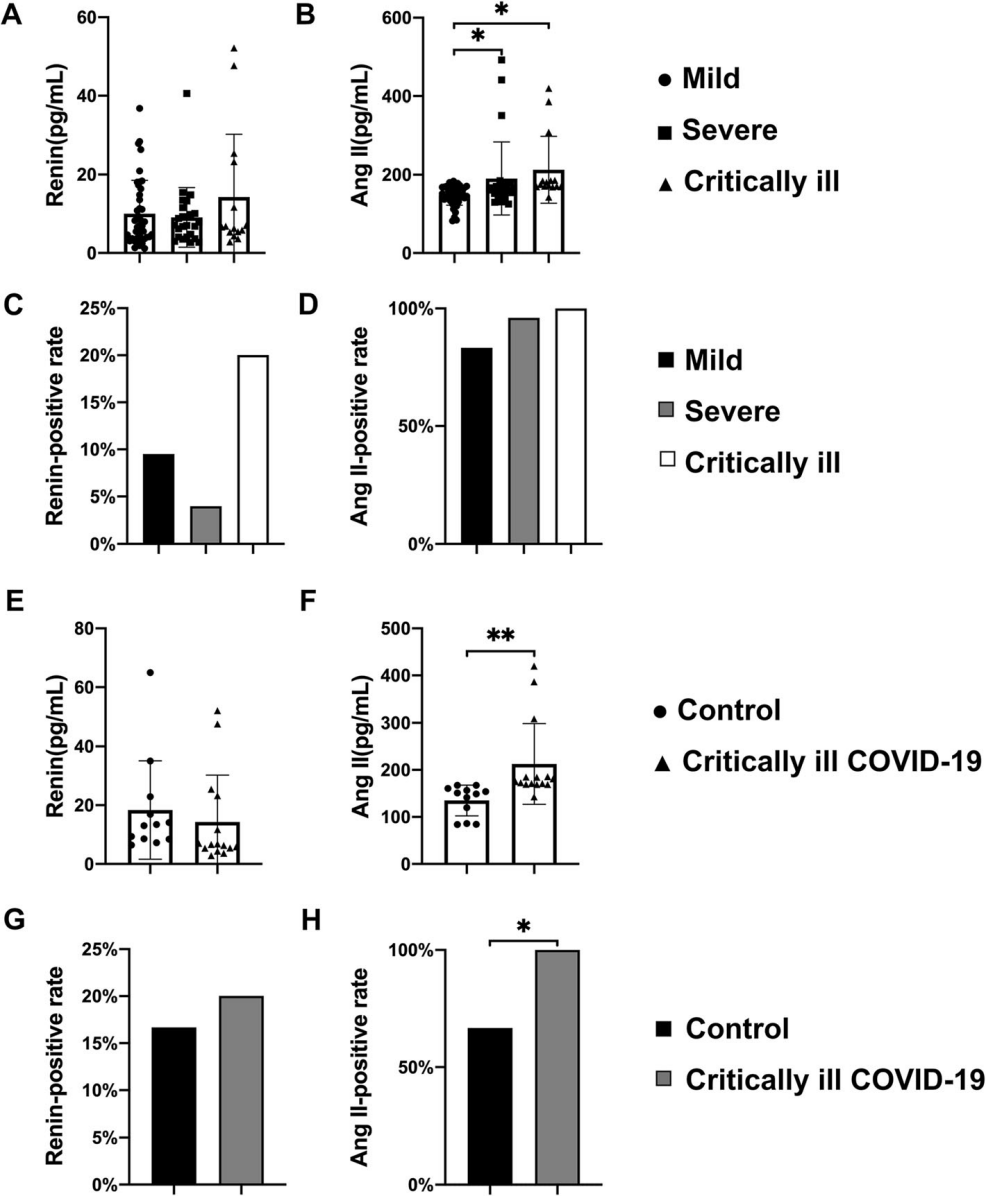
The positive rate and concentration of plasma renin and Ang II in different groups. **a** There were no statistical differences in the concentration of renin in patients with different severity of COVID-19. **b** The plasma Ang II concentration in the critically ill COVID-19 cases and severe COVID-19 cases was significantly higher than that of the mild COVID-19 cases. **c**, **d** The renin and Ang II positive rate in the three groups showed no statistical difference. **e** There was no statistical difference in the concentration of renin in patients with critically ill COVID-19 compared with the control. **f** The plasma Ang II concentration in the critically ill COVID-19 cases was significantly higher than that of control. **g** The renin positive rate in the two groups showed no statistical difference. **h** The plasma Ang II positive rate in the critically ill COVID-19 cases was significantly higher than that of control. Control: critically ill non-COVID-19 cases. *P < 0.05, **P < 0.01

Partial SARS-CoV-2 patients (12.2%) showed elevation of renin content than normal range. There were no statistical differences in renin among the mild, severe, and critically ill COVID-19 patients and control (Fig. 1). These indicated that plasma Ang II elevation was closely related to the SARS-CoV-2 infection, which may be triggered by the interaction between S protein and ACE2 [1, 2]. This played important roles in COVID-19 progression. Previous study showed that subcutaneous Ang II infusion using osmotic pumps for 3 days led to decline of oxygenation and obvious pulmonary injuries after infusion of Ang II for 1 week [3]. For the mechanism, Ang II could promote apoptosis and skeleton reconstruction of pulmonary microvascular endothelial cells, hampering pulmonary microvascular endothelial barrier and the subsequent elevation of pulmonary exudate. This was extremely similar with the quarantine period and pathological manifestations of COVID-19 [4, 5]. Previous study [3] indicated that IL-22 could attenuate Ang II-induced pulmonary injury through modulating JAK2/STAT3 signaling pathway, which may provide new options for treating COVID-19. Therefore, elevation of Ang II triggered by interaction between ACE2 and S protein of SARS-CoV-2 may be important pathogenic factors for critically ill COVID-19 patients.

## Data Availability

The datasets used and analyzed during the current study are available from the corresponding author on reasonable request.

## Abbreviations

SARS-CoV-2: Severe acute respiratory syndrome coronavirus 2
COVID-19: Coronavirus disease 2019
Ang II: Angiotensin II
RAS: Renin angiotensin system
ACE2: Angiotensin-converting enzyme 2

## References

[1] Wan Y, Shang J, Graham R, Baric RS, Li F (2020). Receptor recognition by the novel coronavirus from Wuhan: an analysis based on decade-long structural studies of SARS coronavirus. J Virol.

[2] Khan A, Benthin C, Zeno B, Albertson TE, Boyd J, Christie JD (2017). A pilot clinical trial of recombinant human angiotensin-converting enzyme 2 in acute respiratory distress syndrome. Crit Care.

[3] Wu Z, Hu Z, Cai X, Ren W, Dai F, Liu H (2017). Interleukin 22 attenuated angiotensin II induced acute lung injury through inhibiting the apoptosis of pulmonary microvascular endothelial cells. Sci Rep.

[4] Busse LW, Chow JH, McCurdy MT, Khanna AK (2020). COVID-19 and the RAAS-a potential role for angiotensin II?. Crit Care.

[5] Wu Z, Liu H, Ren W, Dai F, Chang J, Li B (2016). VE-cadherin involved in the pulmonary microvascular endothelial cell barrier injury induced by angiotensin II through modulating the cellular apoptosis and skeletal rearrangement. Am J Transl Res.

